# The Prevalence of Iron and Vitamin D Deficiencies in Pediatric Patients With Inflammatory Bowel Disease in Bahrain

**DOI:** 10.7759/cureus.37074

**Published:** 2023-04-03

**Authors:** Hasan M Isa, Fawzeya A Alahmed, Masooma Mohamed, Afaf Mohamed

**Affiliations:** 1 Department of Pediatrics, Arabian Gulf University, Manama, BHR; 2 Department of Pediatrics, Salmaniya Medical Complex, Manama, BHR; 3 Department of Public Health, Ministry of Health, Manama, BHR

**Keywords:** inflammatory bowel disease, iron deficiency anemia (ida), vitamin-d deficiency, bahrain, biochemical markers, micronutrient deficiency, anemia, ulcerative colitis, crohn’s disease, pediatrics

## Abstract

Introduction

Inflammatory bowel diseases (IBD) are chronic diseases that can affect nutrient absorption leading to micronutrient deficiencies and biochemical abnormalities.This study aimed to assess certain serum micronutrients and nutritionally related biochemical markers levels in patients with pediatric IBD and to compare the actual levels and the prevalence of micronutrients deficiencies and biochemical abnormalities between patients with Crohn’s disease (CD) and those with ulcerative colitis (UC).

Methods

A retrospective cross-sectional study reviewing medical records of patients with IBD was conducted in the pediatric department, Salmaniya medical complex, Bahrain, from 1 January 1984 to 31 December 2021. Demographic data and laboratory results related to micronutrients and biochemical markers including full blood count, total protein, albumin, globulin, iron, ferritin, folic acid, vitamin B12, calcium, phosphorous, magnesium, and vitamin D levels were collected upon presentation before starting the treatment. Nutritional deficiencies were compared based on sex, nationality, type of IBD, age at presentation, disease duration, weight at diagnosis, and inflammatory markers levels including erythrocyte sedimentation rate (ESR) and C-reactive protein (CRP).

Results

Of 157 patients with pediatric IBD, 117 (74.5%) were included. Sixty-six (56.4%) patients were males. Sixty-six (56.4%) had CD and 51 (43.6%) had UC. No patient had indeterminant colitis. The mean age at presentation was 10.8±3.8 years. Most patients had one or more micronutrient deficiencies (n=110, 94%). Anemia was a common finding (n=79/116, 68.1%), with iron deficiency anemia (IDA) being predominant. Low iron levels were noted in 64/77 (83.1%) patients with a median of 5.0 (2.0-9.3) µmol/L (normal range, 11.6-31.3); isolated iron deficiency (ID) in 11/18 (61.1%) and IDA in 53/59 (89.8%) patients. Vitamin D deficiency was the second most common (n=45/61, 73.8%). Serum albumin, ferritin, calcium, phosphorous, and magnesium were deficient in 29.2%, 27.8%, 31.7%, 12.5%, and 10%, respectively. One patient had vitamin B12 deficiency while none had folate deficiency. Patients with CD had significantly lower serum iron (5.4±5.6 versus 8.1±6.09 µmol//L, p=0.02) and lower serum protein (71.7±8.7 versus 75.4±9.9 g/L, p=0.043) but higher serum ferritin (45 (19-110.2) versus 21.3 (10.3-51.2) µg/L, p=0.046) compared to those with UC. Elevated ESR was noted in 62/105 (59.1%) patients while high CRP was found in 67/104 (64.4%). Patients with low iron had higher ESR (28 (17-47) versus 14 (10-33) mm/h, p=0.028) and higher CRP (13.3 (1.6-42) versus 1.8 (0.9-4.6) mg/L, p=0.019) levels compared to those with normal levels.

Conclusion

Patients with pediatric IBD are at risk of multiple micronutrient deficiencies and biochemical abnormalities. Iron and vitamin D deficiencies are the most frequent. Patients with CD are more prone to have lower serum iron and protein levels than those with UC. ID was associated with elevated inflammatory markers.

## Introduction

Crohn’s disease (CD), ulcerative colitis (UC), and indeterminate colitis are chronic diseases of the gastrointestinal tract known as inflammatory bowel diseases (IBD) [1-8]. IBD may present at any age [6]. Yet around 10-25% of cases of IBD begin during childhood or adolescence [8,9]. The most common theoretical cause of IBD is genetic which will lead to an immune reaction that can be triggered by environmental factors [10]. In Bahrain, the estimated annual incidence of pediatric CD was 1/10^5^/year (ranging from 0 to 5 patients) with a prevalence of 9.32 patients per 100,000 [11]. For pediatric UC, the estimated annual incidence was 1/10^5^/year (ranging from 0 to 6 patients) while the prevalence was found to be 16.3 patients per 100,000 [12].

Patients with IBD are prone to multiple nutritional deficiencies including macronutrient and micronutrient deficiencies [13-15]. Hypoproteinemia is an example of partially nutritional macronutrient deficiency [13,14]. Yet, micronutrient deficiency includes low vitamins, minerals, and trace elements levels [10,13,14,16,17]. Both macro- and micronutrient deficiencies can be caused by different mechanisms such as reduced oral intake, systemic inflammation, malabsorption, and increased nutrient losses [8,14,16,18]. Diarrhea can cause electrolyte losses such as calcium, magnesium, and zinc [1]. The most frequent micronutrient deficiencies are iron, vitamin D, and zinc [8,17]. Around 75% of children with IBD present with anemia, and iron deficiency anemia (IDA) is the most common type [8]. Vitamin D and calcium are important for bone health and their deficiency can lead to osteopenia and osteoporosis, especially in patients who received steroids for a long duration [19]. Furthermore, the level of vitamin D has been shown to have an impact on the relapse of IBD [20]. Zinc deficiency can lead to prolonged diarrhea, delayed wound healing, and increased susceptibility to infections [3].

Micronutrient deficiencies are seen more commonly in patients with CD as it mainly involves the terminal ileum and the colon [7,10,11,14,15,17]. Patients with a CD that is complicated with fistula, strictures, and surgical resection in small bowels, or during active disease are more prone to vitamin B12, folic acid, and fat-soluble vitamin deficiencies [10,14]. For example, vitamin D is absorbed in the jejunum and requires the presence of bile acids; accordingly, factors like resection of the small bowel, steatorrhea, or the loss of protein may interfere with its absorption [21-24]. Therefore, it is common to see vitamin D deficiency in patients with CD who had small bowel resection [14].

Despite the advances in the treatment of IBD, micronutrient deficiencies are still frequently seen [14]. The prevalence of macronutrient and micronutrient deficiencies in children with IBD at diagnosis varies widely and it depended on the study population and the criteria used to define these deficiencies. In a recent study, the prevalence of anemia was 57%, iron (56%), ferritin (39%), vitamin A (25%), vitamin D (22%), copper (17%), zinc (10%), selenium (10%), vitamin E (5%), vitamin B12 (2%), and red blood cell folate (1%) [8]. IBD treatment agents may also affect the absorption of micronutrients [12]. Medications such as glucocorticoids inhibit the absorption of calcium, phosphorus, and zinc and may impair vitamins C and D metabolism [25]. Sulfasalazine and methotrexate cause folate deficiency by inhibition of dihydrofolate reductase and cellular uptake [26]. Cholestyramine can affect the absorption of fat-soluble vitamins [27]. Moreover, long-term parenteral nutrition might cause any type of micronutrient deficiency, commonly vitamins A, D, E, zinc, copper, and selenium deficiency, if not provided in sufficient quantities [27]. On the other hand, enteral nutrition (EN) can induce remission of the disease, especially in children with CD with lower cost and fewer side effects compared to parenteral nutrition [3]. Therefore, EN is considered the treatment of choice as it can correct nutritional deficiencies and resume growth if it has been accepted by the child and the family [3]. Hence, it is important to assess the micronutrient levels and correct the deficiencies as the prognosis of IBD can be determined by nutritional status [8,20]. Micronutrient deficiencies have significant implications on the outcomes of patients with IBD, particularly in those with anemia which can lead to lower quality-of-life scores along with cognitive dysfunction [28,29].

Studies about micronutrient deficiencies in children with IBD are limited [8]. To date, there are no published studies about micronutrient levels of pediatric IBD coming from the Kingdom of Bahrain. The aim of this study was to assess the actual levels and the prevalence of anemia, micronutrient deficiencies, and nutritionally related biochemical abnormalities in patients with pediatric IBD in Bahrain and to compare these deficiencies between patients with CD and those with UC.

## Materials and methods

Study design and setting

A retrospective cross-sectional study by reviewing all medical records of patients with IBD (CD, UC, or indeterminate colitis) diagnosed in the pediatric department, Salmaniya medical complex (SMC), Bahrain, from 1^ ^January 1984 to 31^ ^December 2021 was conducted.

Study population

All pediatric patients up to 18 years of age with IBD and available results of laboratory investigations related to at least one of the micronutrients or nutritionally related biochemical markers levels at the time of diagnosis were included in the study. Patients who lack the results of these laboratory tests were excluded. Diagnosis of IBD was established using the IBD working group of the European Society of Pediatric Gastroenterology, Hepatology and Nutrition (ESPGHAN) and the North American Society of Pediatric Gastroenterology, Hepatology and Nutrition (NASPGHAN) criteria [30,31].

Data collection

Data about sex, nationality, type of IBD, age at presentation, weight at diagnosis, duration of the illness prior to diagnosis, medical therapies at the time of diagnosis or during the follow-up course, surgical interventions, and follow-up duration were collected.

Results of laboratory investigations related to micronutrients and biochemical markers including full blood count, total serum protein, serum albumin, serum globulin, serum iron, ferritin, folic acid, vitamin B12, calcium, phosphorous, magnesium, and vitamin D levels were collected upon presentation before starting the treatment. In addition, results of the iron profile including transferrin level, transferrin saturation, and total iron binding capacity (TIBC) were gathered. Standard clinical laboratory techniques were used to measure micronutrients and biochemical markers levels. Anemia was defined as hemoglobin level <11g/dL in children six months to five years old, <11.5 g/dL in children five to 11 years old, <12 g/dL in children 12 to 13 years old, <13 g/dL in men, and <12 g/dL in non-pregnant women [1,32,33]. Patients with sickle cell disease or thalassemia were excluded from the analysis of anemia. Iron deficiency (ID) was diagnosed based on low serum iron in the absence of anemia, while iron deficiency anemia (IDA) was defined based on low serum iron with anemia, with or without low serum ferritin [34]. Inflammatory markers like erythrocyte sedimentation rate (ESR) and C-reactive protein (CRP) levels were also collected.

To determine risk factors for micronutrient deficiencies and nutritionally related biochemical markers abnormalities, patients were divided into two groups according to the presence or absence of the two most common micronutrient deficiencies. Both groups were compared based on sex, nationality, type of IBD, age at presentation, pre-diagnosis disease duration, weight at presentation, and inflammatory markers levels.

Statistical analysis

Patients' data were analyzed using Statistical Product and Service Solutions (SPSS) (IBM SPSS Statistics for Windows, Version 21.0, Armonk, NY). Frequencies and percentages were calculated for demographic, micronutrient deficiencies, and biochemical markers abnormalities data. Continuous variables were presented as mean ± standard deviation (SD) or median and interquartile range (IQR) according to the distribution’s normality. Fisher’s exact test or Pearson’s chi-square was used to compare categorical variables while the Mann-Whitney U test or Student t-test was used to compare continuous variables. The confidence interval was set at 95%. P values <0.05 were considered statistically significant.

Ethical clearance

This study was conducted in accordance with the principles of the Helsinki Declaration of 1975 (revised 2013), and it was ethically approved by the secondary care medical research subcommittee, Salmaniya Medical Complex, Government hospitals, Kingdom of Bahrain (IRB number: 76120521).

## Results

Out of 157 patients diagnosed with pediatric IBD during the study period, 117 (74.5%) patients had the results of laboratory tests available. Despite that, they had laboratory tests performed at the time of presentation, and the remaining 40 (25.6%) patients were excluded due to the unavailability of laboratory results. Demographic data and clinical characteristics of the included patients are shown in Table 1.

**Table 1:** Demographic data and clinical characteristics of patients with pediatric inflammatory bowel disease (n=117). Data presented as number (%), mean ± standard deviation (SD), or median and interquartile range (IQR). ^*^Crohn’s disease, ^†^ulcerative colitis, ^‡^confidence interval. All the percentages were calculated as column percentages. P-values are resulted from ^a^Fisher’s exact test, ^b^Pearson Chi-square, ^c^Mann-Whitney U test, ^d^Students t-test.

Demographic data	Total (n=117)	CD^*^ (n=66)	UC^†^ (n=51)	P value (CI^‡^)
Sex				0.003^a^
Male	66 (56.4)	45 (68.2)	21 (41.2)	
Female	51 (43.6)	21 (31.8)	30 (58.8)	
Nationality				0.578^a^
Bahraini	101 (86.3)	58 (87.9)	43 (84.3)	
Non-Bahraini	16 (13.7)	8 (12.1)	8 (15.7)	
Age at presentation (y)				0.173^b^
0-4.9	9 (7.7)	3 (4.5)	6 (11.8)	
5-9.9	34 (29.0)	17 (25.7)	17 (33.3)	
10-14.9	63 (53.8)	41 (62.1)	22 (43.1)	
15-18	11 (9.4)	5 (7.6)	6 (11.8)	
Pre-diagnosis disease duration (m), median (IQR), (n=90)	3.0 (0.5-10)	3.0 (0.5-8.5)	2.0 (0.5-12.0)	0.990^c^
Weight at diagnosis (kg), median (IQR), (n=66)	30.1 (22.8-39.3)	32.5 (25.0-45.9)	29.0 (20.5-33.0)	0.042^c^
Medications used (n=100)				
Azathioprine	70/98 (71.4)	40/55 (72.7)	30/43 (69.8)	0.748^a^
Folic acid	65/99 (65.7)	34/55 (61.8)	31/44 (70.5)	0.369^a^
Mesalazine	63/98 (64.3)	26/55 (47.3)	37/43 (86.0)	<0.001^a^
Prednisolone	62/100 (62.0)	34/55 (61.8)	28/45 (62.2)	0.967^a^
Omeprazole	58/99 (58.6)	27/55 (49.1)	31/44 (70.5)	0.032^a^
Iron supplementation	46/98 (46.9)	20/55 (36.4)	26/43 (60.5)	0.018^a^
Biological therapy	43/100 (43.0)	26/57 (45.6)	17/43 (39.5)	0.684^a^
Calcium supplementation	30/98 (30.6)	15/55 (27.3)	15/43 (34.9)	0.417^a^
Vitamin D supplementation	32/98 (32.7)	19/55 (34.5)	13/43 (30.2)	0.651^a^
Multivitamins supplementation	27/99 (27.3)	15/55 (27.3)	12/44 (27.3)	1.000^a^
H2 blockers	6.0/98 (6.1)	1.0/55 (1.8)	5.0/43 (11.6)	0.044^a^
Ursodeoxycholic acid	6.0/98 (6.1)	0.0/55 (0.0)	6.0/43 (13.95)	0.004^a^
Fat-soluble vitamins (ADEK) supplementation	4.1/98 (4.08)	0.0/55 (0.0)	4.0/43 (9.3)	0.021^a^
Vitamin B12 supplementation	2.0/98 (2.04)	0.0/55 (0.0)	2.0/43 (4.7)	0.106^a^
Follow-up duration up to 18 y (m), mean±SD	7.2±3.6	6.6±3.1	51±4.0	0.072^d^ (-2.6-0.11)

Most of the patients were males (n=66, 56.4%) and Bahraini (n=101, 86.3%), while 16 (13.7%) were non-Bahraini (five were from India, three from Syria, two from Egypt, two from Pakistan, and one from Morocco, Qatar, Lebanon, and Sweden each). Sixty-six (56.4%) patients had CD and 51 (43.6%) had UC. None of the patients had a diagnosis of indeterminant colitis. The CD was more in males (68.2% versus 41.2%) while UC was more in females (58.8% versus 31.8%), p=0.003. The mean age at diagnosis was 10.8±3.6 years. Six patients (5.1%) had an early-onset disease below five years of age at the time of diagnosis. The mean age at the time of blood collection was 10.79±3.75 years. The median pre-diagnosis disease duration was three (0.5-10) months. Patients with UC had lower median weight (29.0 (20.5-33.0) kg) compared to those with CD (32.5 (25.0-45.9) kg), p=0.042. One patient with UC had concurrent sickle cell disease which was excluded from the analysis of anemia prevalence. No patient had thalassemia. Patients with UC had longer follow-up duration (9.0±8.9 versus 4.8±6.0, p=0.024) compared to those with CD.

The most frequent medications used were azathioprine, folic acid, mesalazine, and prednisolone. Patients with UC were treated more frequently with mesalazine (86% versus 47.3%, p<0.001), omeprazole (70.5% versus 49.1%, p=0.032), iron supplementation (60.5% versus 36.4%, p=0.018), H2 blockers (11.6% versus 1.8%, p=0.044), ursodeoxycholic acid (13.9% versus 0.0%, p=0.004), and fat-soluble vitamins (ADEK) (9.3% versus 0.0%, p=0.021) compared to those with CD. Fat-soluble vitamins were given after the blood collection and as a general support in four patients with UC who had an associated primary sclerosing cholangitis. ADEK was given as 1 mL daily for infants less than one year and 2 mL daily for older children, folic acid 5 mg daily, iron 6 mg per kilogram per day, calcium 250 mg daily (<1 year), 700 mg daily (1-3 years), 1000 mg daily (4-8 years) and 1300 mg daily (9-18 years), vitamin D 400 IU daily, multivitamins 5 mL daily, and vitamin B12 0.5 mcg daily (<1 year), 0.9 mcg daily (1-3 years), 1.2 mcg daily (4-8 years), and 1.8 mcg daily (>9 years) supplementations. Biological therapy was used in 43/100 (43%) patients (27 patients received adalimumab alone, 10 received infliximab alone, four received both types of biologics, and two patients received the non-specific type of biologic therapy). Three patients received exclusive enteral nutrition therapy. None of the patients required intravenous iron therapy or total parenteral nutrition and none agreed to nasogastric tube feeding. Eleven patients required packed red blood cell transfusions. Twenty-five (21.4%) patients underwent gastrointestinal-related surgeries (colectomy (n=11, total in nine and subtotal in three), fistula repair (n=4), inguinal hernia repair (n=3), polypectomy (n=2), ileostomy (n=2), gastrostomy and diaphragmatic hernia repair (n=1, each)). These surgeries were performed after the disease diagnosis.

**Figure 1:**
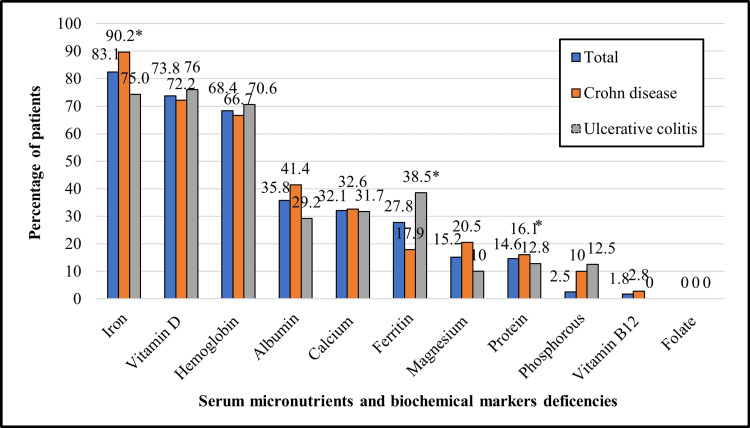
Serum micronutrient deficiencies and biochemical abnormalities in patients with pediatric inflammatory bowel disease. *serum iron (p=0.03), ferritin (p=0.046), protein (p=0.043).

The majority of patients had one or more micronutrient deficiencies or biochemical abnormalities (n=110, 94.02%) while only seven (5.98%) patients had completely normal tested levels. Frequencies of micronutrient deficiencies and biochemical abnormalities are shown in Figure 1.

Anemia was the most common finding (n=79/116, 68.1%), with IDA being predominant. Low iron levels were noted in 64/77 (83.1%) patients with a median iron level of 5.0 (2.0-9.3) µmol/L (normal range, 11.6-31.3); isolated ID in 11/18 (61.1%) and IDA in 53/59 (89.8%) patients. Data on hemoglobin, protein, albumin, and calcium levels were normally distributed while data about iron, ferritin, folate, vitamin B12, phosphorous, magnesium, and vitamin D were not normally distributed. A comparison of laboratory parameters between patients with CD and those with UC is shown in Table 2.

**Table 2:** Comparison of serum micronutrients and biochemical markers levels between patients with Crohn’s disease and those with ulcerative colitis. Data are presented as number (%), mean ± standard deviation, or median (interquartile range). P values are resulted from ^a^Student t test or ^b^Mann-Whitney U test. *Inflammatory bowel disease, ^†^Confidence interval.

Laboratory parameters	Normal values	IBD* total	Low/Total n (%)	Crohn's disease	Low/Total n (%)	Ulcerative colitis	Low/Total n (%)	P value (CI^†^)
Hemoglobin (g/dL)	11-14.5	10.8±2.5	79/116 (68.1)	10.8±2.6	44/66 (66.7)	10.9±2.3	35/50 (70.0)	0.826^a^ (-1.0-0.8)
Protein (g/L)	64-82	73.4±9.4	15/103 (14.6)	71.7±8.7	9.0/56 (16.1)	75.4±9.9	6.0/47 (12.8)	0.043^a^ (-7.4-(-0.1))
Albumin (g/L)	35-52	36.4±7.9	38/106 (35.8)	35.3±8.2	24/58 (41.4)	37.8±7.4	14/48 (29.2)	0.099^a^ (-5.6-0.5)
Iron (µmol/L)	11.6-31.3	5.0 (2.0-9.3)	64/77 (83.1)	4.0 (2.0-7.0)	37/41 (90.2)	5.5 (3.0-11.5)	27/36 (75)	0.03^b^
Ferritin (µg/L)	16-323	29.1 (12.8-77.3)	15/54 (27.8)	45 (19-110.2)	5.0/28 (17.9)	21.3 (10.3-51.2)	10/26 (38.5)	0.046^b^
Folate (nmol/L)	7-28.1	28.1 (19.2-42.2)	0.0/45 (0.0)	29 (21.6-41.7)	0.0/29 (0.0)	23.8 (17.9-36.9)	0.0/16 (0.0)	0.462^b^
Vitamin B12 (pmol/L)	133-675	290 (214.5-398)	1.0/57 (1.8)	318.5 (224.5-382)	1.0/36 (2.8)	250 (213-412)	0.0/21 (0.0)	0.691^b^
Calcium (nmol/L)	2.2-2.7	2.24±0.16	27/84 (32.1)	2.2±0.2	14/43 (32.6)	2.3±0.1	13/41 (31.7)	0.262^a^ (-0.1-0.0)
Phosphorous (mmol/L)	0.81-1.45	1.4 (1.2-1.5)	2.0/80 (2.5)	1.4 (1.3-1.5)	4.0/40 (10.0)	1.4 (1.1-1.5)	5.0/40 (12.5)	0.382^b^
Magnesium (mmol/L)	0.74-1.0	0.8 (0.8-0.9)	12/79 (15.2)	0.8 (0.8-0.9)	8.0/39 (20.5)	0.8 (0.8-0.9)	4.0/40 (10)	0.729^b^
Vitamin D (nmol/L)	>50	35 (24-52)	12/79 (15.2)	32.4 (24.5-60.5)	26/36 (72.2)	37 (24-41)	19/25 (76)	0.493^b^

Patients with CD had significantly lower serum iron (5.4±5.6 versus 8.1±6.09 µmol/l/L, p=0.03) and lower serum protein (71.7±8.7 versus 75.4±9.9 g/L, p=0.043) but higher serum ferritin (45 (19-110.2) versus 21.3 (10.3-51.2) µg/L, p=0.046) compared to those with UC. Despite those patients with CD had lower levels of iron compared to those with UC, the use of iron supplements was higher among patients with UC (p=0.018), see Table 1. There was no significant difference in other parameters. Detailed iron profile data was available in 44 (37.6%) patients. The mean transferrin level was 2.5±0.74 g/L (normal range, 2.15-3.65); 14 (31.8%) had low, 29 (65.9%) had normal, and one (2.3%) had high levels. The median transferrin saturation was 8.5% (IQR, 5.0-14.8) (normal range, 15-33); low in 33 (75%) and normal in 11 (25%) patients. The mean TIBC was 63.4±26.4 µmol/L (normal range, 46-80); it was high in nine (20.5%) and normal in 35 (79.5%) patients. Serum folate levels were normal in all tested patients. One (1.8%) patient with CD had vitamin B12 deficiency and this patient was anemic and had an associated iron deficiency.

Vitamin D deficiency was the second most frequent micronutrient deficiency (n=45/61, 73.8%). Serum calcium was deficient in 32.1% (n=27/84) of the patients while hypomagnesemia was in 15.2% (n=12/79). In terms of pre-diagnosis disease duration, no significant difference was found between patients with hypoalbuminemia (2.0 (0.3-5.0) months) and those without (3.0 (0.5-12) months), p=0.458. Sixty-two out of 105 (59.1%) patients had high ESR and 67 of 104 (64.4%) had high CRP. A comparison between patients with the two most common micronutrient deficiencies (iron and vitamin D deficiencies) and those without is shown in Table 3.

**Table 3:** Relationship between demographic data, serum iron, and vitamin D levels in patients with pediatric inflammatory bowel diseases. Data presented as number (%) or mean ± SD or median (IQR). ^*^confidence interval, ^†^standard deviation, ^‡^interquartile range. P values are resulted from ^a^Fisher’s exact test, ^b^Student t-tests, ^c^Pearson Chi-square, ^d^Mann Whitney U test. All the percentages were calculated as column percentages.

Variable	Iron level, n=77		P value (CI*)	Vitamin D level, n=61		P value (CI*)
	Normal, n=13 (16.9)	Low, n=64 (83.1)		Normal, n=16 (26.2)	Low, n=45 (73.8)	
Sex			0.062^a^			0.562^a^
Male	4 (30.8)	40 (62.5)		10 (62.5)	23 (51.1)	
Female	9 (69.2)	24 (37.5)		6 (37.5)	22 (48.9)	
Nationality			1.000^a^			0.224^a^
Bahraini	11 (84.6)	52 (81.3)		12 (75)	40 (88.9)	
Non-Bahraini	2 (15.4)	12 (18.8)		4 (25)	5 (11.1)	
Type of inflammatory bowel disease			0.126^a^			0.777^a^
Crohn’s disease	4 (30.8)	37 (57.8)		10 (62.5)	26 (57.8)	
Ulcerative colitis	9 (69.2)	27 (42.2)		6 (37.5)	19 (42.2)	
Age at presentation (y), mean ± SD	10.8±4.3	10.4±3.6	0.661^b ^(-1.7-2.7)	8.9±3.1	10.5±3.7	0.147^b^ (-3.4-0.4)
Age group at presentation (y)			0.844^c^			0.217^c^
0-4.9	1 (7.7)	6 (9.4)		3 (18.8)	4 (8.9)	
5-9.9	3 (23.1)	22 (34.4)		8 (50)	14 (31.1)	
10-14.9	8 (61.5)	31 (48.4)		5 (31.2)	24 (53.3)	
15-18	1 (7.7)	5 (7.8)		0 (0.0)	3 (6.7)	
Pre-diagnosis disease duration (m), median (IQR), (n=90)	5.0 (0.2-24)	2.0 (0.5-5.0)	0.742^d^	2.0 (0.3-3.0)	2.3 (0.4-10)	0.431^d^
Weight at diagnosis (kg), median (IQR), (n=66)	29.7 (15-40)	30 (23-34)	0.922^d^	24.8 (21.6-30)	31.2 (24-40)	0.240^d^
Erythrocyte sedimentation rate (mm/h), median (IQR), (n=105)	14 (10-33)	28 (17-47)	0.028^d^	28 (15-45)	22 (15-50)	0.915^d^
C-reactive protein (mg/L), median (IQR), (n=104)	1.8 (0.9-4.6)	13.3 (1.6-42)	0.019^d^	11.4 (1.3-47.6)	10 (1.3-48.2)	0.714^d^

Patients with low iron had higher ESR (28 (17-47) versus 14 (10-33) mm/h, p=0.028) and higher CRP (13.3 (1.6-42) versus 1.8 (0.9-4.6) mg/L, p=0.019) levels compared to those with normal levels. There was no significant relation found between serum ferritin levels and elevated ESR (low ferritin=8/15 (53.3%) versus normal=26/38 (68.4%), p=0.351) or elevated CRP levels (low ferritin=7/15 (46.7%) versus normal=27/38 (71.1%), p=0.120).

## Discussion

Anemia in patients with IBD has received little attention from clinicians in terms of diagnosis and treatment, despite that overt or occult gastrointestinal bleeding is a common symptom in this group of patients [16]. In patients with either CD or UC, a drop in hemoglobin is expected with almost every flare-up of their disease [16]. This study showed a high prevalence of anemia in patients with pediatric IBD at their presentation (68.1%). This is double the prevalence of anemia in school children aged 6-14 years in Bahrain which was reported to be 37% [35]. Also, other studies published from Bahrain by Alalawi et al. reported a prevalence of anemia of 40.5% in nine months old while Alhaddad reported an anemia prevalence of 30% in three-year-old children [36,37]. In these studies, the average hemoglobin level in Bahrain was not recorded. Yet, Rempel et al. study from Canada [8] and Wiskin et al. from the United Kingdom [9] reported a comparable prevalence of anemia in their patients with IBD at the time of presentation, 64% and 75%, respectively. Likewise, a Korean study by Song et al. [1] showed that 62% of their patients with pediatric CD were diagnosed with anemia. In our study, there was no significant difference in the anemia prevalence between patients with CD (66.7%) and those with UC (70%). Ehrlich et al. [13] and Takaoka et al. [15] also found no significant difference in the hemoglobin levels between patients with CD and those with UC. However, in Rempel et al. [8] study, anemia was proportionally higher in patients with CD (61%) versus those with UC (52%). Based on the high prevalence of anemia in this study, we recommend that anemia should be given better consideration in patients with IBD through thorough investigation and proper management.

Anemia can develop secondary to iron, vitamin B12, and folic acid deficiencies [16]. Low iron levels were noted in 83.1% of our patients; 61.1% has isolated ID and 89.8% had IDA. Accordingly, iron deficiency was the most common micronutrient deficiency that was seen in this study. This is almost triple the prevalence of IDA among normal children aged three years from Bahrain which was reported to be 30% [37]. In the context of IBD, IDA and anemia of chronic illness are the preponderate two types of anemia [9,16]. However, IDA is the most prevalent form of anemia in patients with pediatric IBD due to a lack of an adequate amount of iron to form normal red blood cells [1,8,9]. IDA can be caused by dietary restriction, chronic blood loss, and malabsorption of iron in the duodenum secondary to mucosal inflammation and rapid bowel transient time [1,9,10,14,17,38]. Rempel et al. [8] also attributed the high prevalence of IDA in patients with CD to the reduced iron absorption in the proximal jejunum due to the CD activity that affects the area. The prevalence of IDA among preschool children from the Arab Gulf countries ranged from 20% to 67%, while that among school children ranged from 12.6% to 50% [39]. However, Song et al. [1] reported a lower percentage of iron deficiency in south Korean children with CD than that in our study (77%). In the current study, patients with CD had a higher prevalence of ID compared to those with UC (90.2% versus 75%, p=0.03). These findings were comparable to Ehrlich et al. [13] and Motil et al. [40] studies which also showed lower levels of serum iron in children with CD compared to those with UC. Ehrlich et al. [13] reported ID in 88.8% and 76.9% of patients with CD and UC, respectively. Motil et al. [40] reported a mean iron level of 56±25 µg/L in patients with CD versus 86±43 µg/L in those with UC. Yet, Rempel J et al. [8] and Wiskin et al. [9] reported almost equal percentages of IDA in patients with pediatric CD and those with UC, 56% versus 55% and 90% versus 95%, respectively. This could be explained by the role of iron therapy given to patients with UC to treat IDA prior to the diagnosis. Patients with UC usually present with bloody diarrhea compared to those with CD who usually present with recurrent abdominal pain as shown in our previous studies [11,12]. This bloody diarrhea might mandate earlier presentation to healthcare services, earlier diagnosis of IDA, and prompt treatment with iron supplementation. In a systematic review, Fritz et al. [20] recommended prescribing a multivitamin that contains iron, vitamin D, and zinc to all patients with IBD as they are more likely to develop micronutrient deficiencies regardless of their disease activity or phenotype. The findings of our study also support the recommendation of supplementing patients with IBD with iron regardless of the patients' sex, type of IBD, age at presentation, or disease duration; especially those with high inflammatory markers (ESR and CRP). Children with IBD are at a higher risk of multiple micronutrient deficiencies than the general population [13-15,20]. Iron deficiency is common, 95% of patients have been reported to have ID at diagnosis, and up to 70% may continue to have ID two years after diagnosis [20]. This may be due to poor disease control, insufficient oral intake, and/or inadequate supplementation. Iron deficiency may continue despite oral supplementation [20]. Accordingly, we recommend iron supplementation for pediatric patients with IBD along with continuous monitoring of its level.

Ferritin was found to be either normal or high in most of our patients and only 27.8% had low ferritin level while Rempel et al. [8] reported low ferritin in 60% of pediatric patients with UC and in 21% of those with CD. This variation is expected because ferritin is an acute phase reactant and can increase with chronic inflammation, chronic disease, or infection which is the case in patients with IBD [9]. ESR was elevated in 59.1% of our patients and 64.4% had high CRP at presentation. Moreover, high ferritin levels might mask the presence of an underlying functional iron deficiency [9]. Although serum ferritin is the most frequently used test to identify isolated ID as it reflects low iron stores [34], this should not be applied in patients with IBD. Patients with IBD can develop IDA along with anemia of chronic disease [34]. Despite that the distinction of IDA from anemia of chronic disease is straightforward, there is no clear-cut test that diagnoses IDA in the setting of inflammation such as in patients with IBD [34]. Serum ferritin is consensually decreased in IDA, but the ferritin level is increased in the anemia of chronic inflammation, reflecting macrophage iron sequestration [34]. This might explain the lower percentage of low ferritin levels (27.8%) in our patients with ID (83.1%). Therefore, ferritin should not be considered a reliable test for ID in patients with IBD.

Vitamin B12 deficiency is rare in pediatric IBD diagnosis [13]. Unlike patients with UC which usually causes inflammation limited to the colon, vitamin B12 is commonly seen to affect patients with CD [14]. This can be explained by the fact that CD usually affects the terminal ileum causing long-term inflammation and fibrosis which interferes with vitamin B12 absorption resulting in vitamin B12 deficiency [14]. In this study, only one (1.8%) patient with CD had vitamin B12 deficiency and this patient was anemic and had an associated iron deficiency. The low prevalence of vitamin B12 deficiency in our study might be explained by that none of our patients had terminal ileum resection at the time of blood collection and even the resection of part of the terminal ileum does not lead to vitamin B12 deficiency. Similarly, Rempel et al. [8] study reported one (1%) patient with CD to have vitamin B12 deficiency along with two (3%) patients with UC. This low prevalence of vitamin B12 deficiency might be attributed to the fact that the vitamin B12 body stores might be sufficient for many months or even years, and most of the patients with complicated CD underwent limited terminal ileal resections [13]. Motil et al. [40] study showed no significant difference in vitamin B12 levels between children with CD and those with UC. Yet, Kalantari et al. [2] reported a remarkably high proportion of vitamin B12 deficiency in adult patients with mild and moderate to severe UC (35% and 50%, respectively). Fritz et al. stated that screening for vitamin B12 deficiency should be limited to patients with ileal or ileocolonic resection, and those with suspected malabsorption [20]. Based on our study findings and the literature reviewed, we agree with this statement that testing for vitamin B12 deficiency should be limited for patients with extensive ileal resection and those with severe malabsorption. However, Kim et al. [10] and Hwang et al. [14] stated that IBD is a disease that affects malabsorption and it's important to investigate vitamin B 12 deficiency, especially in patients with CD, as steatorrhea, severe ileitis, and terminal ileum resection can affect the absorption.

In the current study, all 45 (38.5%) tested patients for folate showed normal or even high levels, 23 (51.1%) and 22 (48.4%) patients, respectively. Comparably, Ehrlich et al. [13] reported a low prevalence of folate deficiency, 10% and 3.8%, in patients with CD and UC, respectively. However, Song et al. [1] study reported folate deficiency in 40% of their cohort. Folate is an essential micronutrient for deoxyribonucleic acid (DNA) synthesis and methylation reactions of red blood cells [13]. Folate deficiency can lead to macrocytic megaloblastic anemia [14]. Yet, the findings of our study do not support regular folate supplementation. 

The role of vitamin D in calcium homeostasis is well-recognized in pediatric patients with IBD [4,14,41,42]. Nonetheless, the relation between vitamin D deficiency and the etiopathogenesis and activity of IBD is still under investigation [8,43]. The relationship between IBD and vitamin D status is bidirectional. While IBD can cause vitamin D deficiency due to the malabsorption of vitamin D in the ileum and jejunum as well as the poor digestion of lipid-soluble vitamins and the severity of the disease that decreases the patient's exposure to the sunlight as they are limited to bed rest [4], vitamin D deficiency was linked with the IBD disease activity and exacerbation [44]. This might explain the relatively high prevalence of vitamin D deficiency in our patients with IBD. In this study, vitamin D was deficient in 73.8% of patients with IBD being the second most frequent micronutrient deficiency after iron deficiency. Kalantari et al. [2] also reported a higher prevalence of vitamin D deficiency in their pediatric patients with UC (87%). Nonetheless, this prevalence is still considered low compared to that of healthy age-matched counterpart children in Bahrain where vitamin D deficiency prevalence is extremely high (93.4%) [45]. On the contrary, El-Matary et al. [43] study from Canada showed that children with newly diagnosed IBD had significantly lower levels of vitamin D compared to those without (p=0.04). Patients with IBD are at higher risk of vitamin D deficiency due to their low oral intake and poor absorption of vitamin D due to the inflammatory process of the disease, especially during flare-ups [4]. However, many other factors can also affect vitamin D levels [45]. The differences in the vitamin D levels between studies can be explained by the different definitions of normal serum vitamin D levels, variation in the degree of sun exposure, and other environmental factors including clothing, indoor lifestyle, seasonal variation, and environmental pollution [43,45,46]. Our study also showed that vitamin D deficiency was more in patients with UC (76%) compared to those with CD (72.2%), p=0.046. Yet, this is a borderline difference, and it might not be evident had our population been larger. Nonetheless, this finding is similar to Rempel et al. [8] which showed that 28% of patients with UC and 16% of patients with CD had vitamin D deficiency. However, El-Matary et al. [43] found lower vitamin D levels in patients with UC compared to those with CD but this difference was not statistically significant. Sohn et al. [4], Ehrlich et al. [13], and Boot et al. [42] reported similar levels of vitamin D between children with CD and those with UC. Based on our results and other research results, we recommend vitamin D supplementation for patients with IBD.

In the current study, 32.1% of patients were deficient in calcium and 15.2% had hypomagnesemia. Song et al. study [1] also showed that 35% and 10% of children were deficient in calcium and magnesium, respec­tively. These findings can be justified as all 71 children with CD in their study presented with severe low bone mineral density (BMD) [1]. In our study, besides the high prevalence of vitamin D deficiency which can cause hypocalcemia, the second most common symptom in our patients was diarrhea [11,12]. Hypocalcemia can be a result of vitamin D deficiency, as well as small bowel inflammation [14]. Both calcium and magnesium deficiencies in patients with IBD can be caused by gastrointestinal loss and inadequate intake [1]. Moreover, hypomagnesemia has been linked to inadequate dietary intake and diarrhea, this can subsequently affect calcium absorption in the intestine [1,14,18].

Protein-energy undernutrition is one of the forms of undernutrition in patients with IBD [16]. The serum protein level is an important nutritional parameter that can be low in these patients [8]. Hypoproteinemia can be due to poor oral intake or secondary to protein-losing enteropathy [3,8,14]. Protein loss can occur in severe and active IBD [14]. Although oral intake of protein and protein-losing enteropathy were not assessed specifically in our study, both mechanisms might be relevant to our population, especially in patients with CD who presented more with anorexia, recurrent abdominal pain, and chronic diarrhea as shown in our previously published studies [11,12]. In this study, patients with CD were found to have more hypoproteinemia (16.1%) than patients with UC (12.8%), p=0.043. This finding is similar to Motil et al. [40] study which reported significantly lower levels of serum total proteins in children with CD compared to those with UC. Conversely, Takaoka et al. [15] found that patients with CD had higher levels of total serum protein (6.4±0.8 g/dL) compared to those with UC (5.8±0.06 g/dL), p=0.009.

Serum albumin level can be used as a barometer of IBD activity [17]. The current study revealed a high prevalence of hypoalbuminemia (35.8%). Yet, Song et al. [1] showed a higher percentage of hypoalbuminemia (61%) than that of our study. Hypoalbuminemia was more frequent in patients with CD and those with UC in our study, 41.4% in CD and 29.2% in UC, but this difference was not statistically significant (p=0.225). Abdul Aziz et al. [5] and Motil et al. [40] also reported lower levels of serum albumin in children with CD compared to those with UC. Patients with CD are more prone to develop hypoalbuminemia as they usually present more with anorexia, recurrent abdominal pain, and chronic diarrhea which can lead to lower oral intake and higher protein loss [11,12]. Moreover, patients with CD usually have longer disease duration prior to diagnosis compared to those with UC, as shown in this study. However, Takaoka et al. [15] found that patients with UC had a higher percentage of hypoalbuminemia (83.3%) compared to those with CD (54.5%), but this difference was not statistically significant (p=0.053).

In the current study, patients with low iron level had significantly higher inflammatory markers including ESR (p=0.028) and CRP (p=0.019) levels. Song et al. [1] and Benjamin et al. [47] studied patients with CD and found that biomarker parameters such as hemoglobin, serum iron, folate, total protein, albumin, calcium, magnesium, and zinc were significantly lower in patients with active disease compared to those in remission. However, in patients with active disease, plasma concentrations of many micronutrients should be interpreted with caution as their levels will not be representative of the actual body stores [16]. Nonetheless, Ehrlich et al. [13] found no association between micronutrient status and disease activity. Apart from potassium level, Kalantari et al. [2] also reported no significant differences in all laboratory parameters according to disease severity in patients with UC.

Like any other retrospective study, this study was limited by missing data about patients’ demography, results of laboratory investigations, or medications used in some patients. Accordingly, only patients with available data about laboratory test results were included in the analysis while those missing relevant data we excluded. This selection bias was unavoidable as the aim of the study was to calculate the valid percentage for each nutrient deficiency to avoid underestimation of the true prevalence of each deficiency. Moreover, to the best of our knowledge, no specific hemoglobin norm was published in our country. However, in our region, El-Hazmi and Warsy from Saudi Arabia published hematological references for Saudi healthy children and adolescents aged 1-15 years [33]. Their hemoglobin level was ranging between 11.3 and 14.2 g/dL. Nonetheless, most of the studies reviewed were adopting the world health organization (WHO) norm [32]. In this study, we followed the hemoglobin range of 11-14.5 g/dL. In addition, serum iron level was used to diagnose IDA in this study. Serum iron alone is considered as an unreliable marker as it can be affected by several factors including inflammatory processes, diurnal variation, malignancy, and menstrual blood loss [8]. Vitamin D levels also vary according to the degree of sun exposure, clothing, dietary intake, environmental pollution, and seasonal variation which makes checking it at a single point in time might not reflect real vitamin D status [43,45]. Yet, the effect of seasonal variation on vitamin D levels might not be significant in children with IBD as most of those with severe disease are limited to bed rest and indoor confinement [4]. Other biomarkers and micronutrients such as zinc, copper, selenium, and vitamin (A, B complex, C, and E) levels were not included in this study. Also, dual x-ray absorptiometry (DEXA) results, oral protein intake, and protein-losing enteropathy were not assessed in this study. Furthermore, data about the disease activity and disease severity scores were not available. Despite these limitations, this study is important being the first study to tackle the prevalence of anemia, nutritionally related biochemical abnormalities, and micronutrient deficiencies in patients with pediatric IBD from Bahrain, which can form a foundation for future studies.

## Conclusions

This study showed that iron and vitamin D deficiencies are the most frequent micronutrient deficiencies found in patients with pediatric CD and UC. Moreover, patients with CD showed lower serum iron and protein levels compared to those with UC. Iron deficiency was associated with elevated inflammatory markers. Further studies to assess the effect of micronutrient deficiencies on IBD pathogenesis and on patients’ clinical symptoms and signs are specifically needed. Furthermore, evaluating the long-term impact of using IBD medications on micronutrient levels is warranted. Studies are also required to explore the role of prescribing multivitamins and other micronutrients in altering the disease activity and the quality of life in this group of patients.
